# Generating Virtual Patients by Multivariate and Discrete Re-Sampling Techniques

**DOI:** 10.1007/s11095-015-1699-x

**Published:** 2015-05-21

**Authors:** D. Teutonico, F. Musuamba, H. J. Maas, A. Facius, S. Yang, M. Danhof, O. Della Pasqua

**Affiliations:** Division of Pharmacology, Leiden Academic Centre for Drug Research, Leiden, The Netherlands; Clinical Pharmacology Modelling & Simulation, GlaxoSmithKline, Stockley Park, Middlesex UK; Department of Pharmacometrics, Nycomed GmbH, Constance, Germany; Clinical Pharmacology & Therapeutics, University College London, BMA House, Tavistock Square, London, WC1H 9JP UK

**Keywords:** clinical trial simulations, covariate effect, demographics, drug development, inclusion and exclusion criteria, multivariate distribution, re-sampling

## Abstract

**Purpose:**

Clinical Trial Simulations (CTS) are a valuable tool for decision-making during drug development. However, to obtain realistic simulation scenarios, the patients included in the CTS must be representative of the target population. This is particularly important when covariate effects exist that may affect the outcome of a trial. The objective of our investigation was to evaluate and compare CTS results using re-sampling from a population pool and multivariate distributions to simulate patient covariates.

**Methods:**

COPD was selected as paradigm disease for the purposes of our analysis, FEV1 was used as response measure and the effects of a hypothetical intervention were evaluated in different populations in order to assess the predictive performance of the two methods.

**Results:**

Our results show that the multivariate distribution method produces realistic covariate correlations, comparable to the real population. Moreover, it allows simulation of patient characteristics beyond the limits of inclusion and exclusion criteria in historical protocols.

**Conclusion:**

Both methods, discrete resampling and multivariate distribution generate realistic pools of virtual patients. However the use of a multivariate distribution enable more flexible simulation scenarios since it is not necessarily bound to the existing covariate combinations in the available clinical data sets.

**Electronic supplementary material:**

The online version of this article (doi:10.1007/s11095-015-1699-x) contains supplementary material, which is available to authorized users.

## Introduction

Whilst simulations have existed for many years as a statistical technique, their use as tool to evaluate treatment response in clinical trials has become possible thanks to the integration of disease progression and pharmacokinetic-pharmacodynamic (PKPD) models. From a drug development perspective, clinical trial simulation (CTS) became a useful tool to support decision making and reduce clinical trial failure (1–3). Among the numerous possible applications, CTS has been used to characterise the behaviour of a biological systems, explore drug properties as well as understand response in different populations. It can also be used to rank or prioritise options for a drug development program, thereby providing an integrated overview of potential designs and outcomes to relevant stakeholders (e.g., clinical experts, regulatory authorities). Moreover, it offers the opportunity to test different “what if” scenarios from first-time-in-human studies throughout post-marketing studies in phase IV (4).

**Fig. 1:**
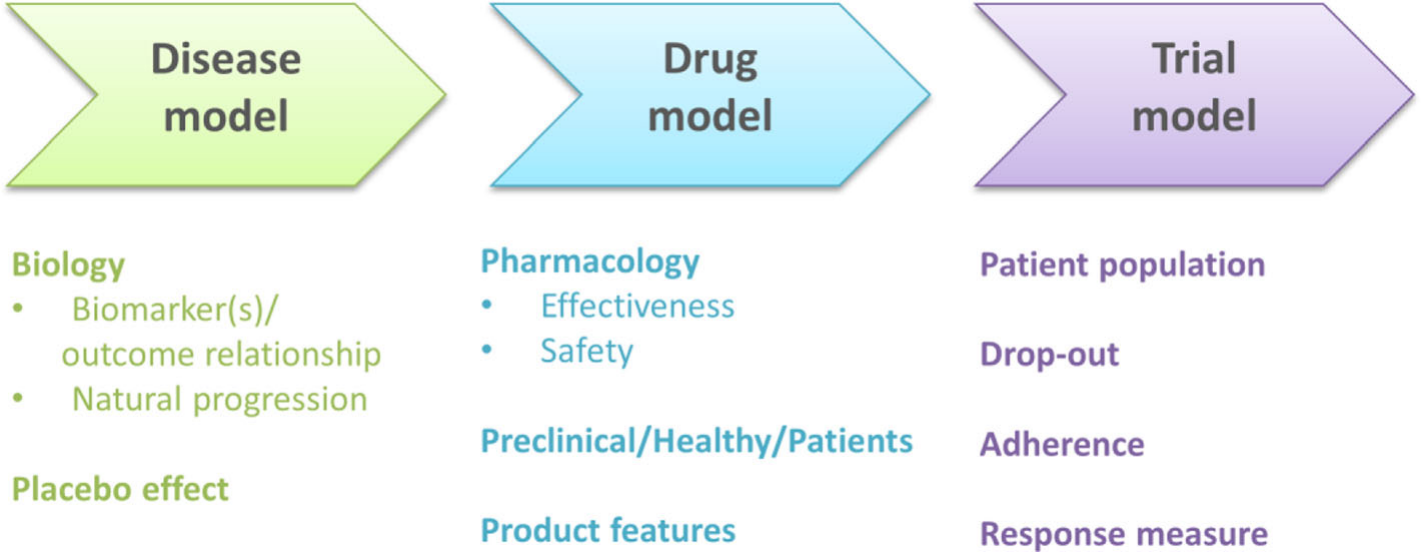
Components and factors to be considered in a clinical trial simulation (adapted from Gobburu and Lesko) (7). Little focus has been given to the trial model. Given that the objective of a CTS exercise is to evaluate how multiple interacting factors in a specific scenario affect the outcome of the study, it is essential to properly simulate patient populations which reflect individual characteristics in a realistic manner, including an evaluation of the impact of individuals who do not meet inclusion/exclusion criteria.

The technical definition of CTS includes the generation of a response for a virtual subject by reproducing the trial design, the disease progression, the drug and the patient’s behaviour using mathematical models and numerical methods (5, 6) (Fig. 1). Even though much attention has been given to disease and drug models, limited efforts have been made to ensure the accurate evaluation of trial design factors, patient behaviour and individual characteristics in clinical trial simulations. The trial execution model represents the design variables of interest in a simulation exercise (e.g. dosing regimens, selection criteria, stratification rules, study duration); the patient’s behaviour comprises those factors that determine the trial execution features, such as adherence and missing records. Individual characteristics include demographic, clinical and physiological measures that altogether describe individual patients in the target population.

Since the objective of a CTS exercise is to evaluate how multiple interacting factors in a specific scenario affect the outcome of the study, it is essential to properly simulate patient populations which reflect individual characteristics in a realistic manner, including an evaluation of the impact of individuals who do not meet inclusion/exclusion criteria, as defined for evidence arising from controlled clinical trials. Thus far, in most cases, published literature regarding the use of simulations in clinical pharmacology has been limited to the evaluation of the effect of changes in some specific design factors, such as the study duration, dose or sampling frequency (8–11). By contrast, there is very little work done on the impact of design factors related to patient selection and protocol stratification. Yet, these publications often do not consider the implication of such factors on trial outcome. This is particularly important if one wants to understand the role of physiological or demographic factors as covariates on response parameters and assess the possibility for personalised therapy or dose adjustment (12).

In this context, covariate distribution models play an essential role. They describe patient-specific aspects defining e.g., patient demographic information, baseline disease characteristics, co-morbidity and concomitant medication (13). For each patient, these details may be considered as a vector containing the patient information (e.g., male, 70 kg, smoker, severe disease status, etc.). This information vector is usually associated with the differences between patients in terms of pharmacokinetics and pharmacodynamics, and it is often used to explain the variability on individual parameter values. Consequently, the simulation of covariates for a virtual patient population assumes great importance as the individual vectors of the covariates for each patient will determine the outcome of the simulated study.

Statistically, the approaches used to simulate virtual populations may be divided in nonparametric and parametric simulations. Nonparametric bootstrapping or re-sampling is one of the most straightforward methods for constructing a population of virtual patients, often used to determine the uncertainty on estimated or predicted quantities (14, 15). Using this approach, a pool of patients can be created by randomly selecting patient vectors for inclusion into the virtual pool of patients. This first random selection of individuals can then be refined by applying inclusion–exclusion criteria in order to obtain the desired population. In general, numerous covariates are correlated, and as such these correlations need to be maintained during the creation of virtual patients. For instance, in paediatric patients, age and weight are generally related and any re-sampling technique will need to account for that correlation (16). In this sense, re-sampling methods provide an opportunity to create patients with a realistic combination of covariates. Nevertheless, the technique suffers an important limitation, i.e., the constraint of the observed covariate matrix in the real data (empirical distribution). Consequently, one should be aware of the fact that the simulated population will not include patients with any other combination of covariates other than the one observed in the source data.

Parametric methods, on the other hand, may be used as alternative to re-sampling in order to generate new combinations of vectors or matrices from an existing distribution. The two main methods for generating covariate vectors with this approach are represented by a series of univariate distributions or by using a unique multivariate distribution for the whole population. The advantage of this latter technique consists in accounting for the correlation between covariates in the simulation process, which allows for the simulation of more realistic covariate vectors. Whilst it can be anticipated that a mechanistic or physiologically-based model may be required with increasing number of interacting factors (17, 18), in many cases, these interactions may be defined using existing knowledge or may be extrapolated from an existing population.

Independently from which choices are made, the method for obtaining simulated data sets should be carefully considered and its performances verified afterwards (19). The simulated data sets should resemble reality if the results are meant to reflect real-life conditions. The correlation between covariates can be estimated from real data to ensure that the simulated data closely reflect the underlying pool of patients. Subsequently, the distribution of the simulated data should be verified to confirm they resemble what is being simulated. This can be done, for example by using summary measures for the covariate distributions, Kaplan–Meier survival curves for survival data or by fitting appropriate regression models.

The objective of this exercise was therefore to compare the use of discrete re-sampling and multivariate normal distribution (MVND) methodologies in the creation of virtual patient populations showing plausible, realistic vector of clinical and demographic characteristics. The MVND methodology developed by Tannenbaum and collaborators (20), which is usually applied to the simulation of continuous variable, was adapted to allow the simulation of categorical data. The approach consists in treating all categorical covariates as if they have continuous values; new covariate values are sampled from a distribution built using the real data and the simulated data are then converted back again into categorical ones. Chronic obstructive pulmonary disease (COPD) was selected as a case study for the comparison of the two methodologies. A previous investigation performed by our group on the progression of COPD has shown that symptoms and severity in this population are closely related to a combination of multiple covariates, continuous as well as categorical ones, including demographics and disease-specific characteristics (21, 22). Moreover, considering the large number of COPD trials failed in the past few years, we anticipate that covariate effect profiling will be critical for the design and evaluation of efficacy trials in this field.

## Methods

As the objective of this exercise was to compare the use of discrete re-sampling and multivariate normal distribution (MVND) methodologies in the creation of virtual patient populations, attention was paid to their performance in generating a plausible, realistic vector of clinical and demographic characteristics. Continuous and categorical covariates were simulated using a real set of COPD patients from the Dutch TI Pharma database. Patient demographics and data set details are reported in Table I. The data set used in the simulation consisted of 3498 patients (32% women), and the covariates included in the analysis were COPD severity, smoking status, gender, weight, height and age. The covariate simulation was performed with R 2.12.0 (23). This patient population was used as empirical distribution for the subsequent steps in our analysis.

**Table I:** Summary of the COPD Patient Demographics (n=3498) Extracted from the TIPharma Database for the Purposes of the Current Analysis

Categorical variables	Number of subjects	%
Gender (males)	476	68
Smoking status		
Smoker	123	32
Ex-smoker	006	57
Non smoker	369	11
Severity		
Mild / moderate	180	34
Severe/very severe	318	66
Continuous variables	Median (range)	
Age (yr)	65 (40, 90)	–
Weight (kg)	75 (35, 183)	–
Height (cm)	170 (135, 208)	–
Baseline FEV1 (L)	1.14 (0.33, 3.18)	

### CTS with Re-Sampling and MVND Patient Generation

Discrete re-sampling was performed using the R package MStoolkit (Mango Solutions, UK). The data set used in the simulations contains only one line per subject, i.e., where applicable multiple time measures were removed and each patient was randomly sampled as a vector of covariates. Each patient could be sampled multiple times in order to obtain pools of patients greater than the original data set, and to introduce variability when an equal number of patients was simulated.

In order to generate continuous and categorical covariates in parallel, a set of pre-defined data manipulation steps is required before the simulations are performed. The MVND methodology was based on the approach described by Tannenbaum *et al.* (20). Briefly, with this technique the continuous and categorical covariates are treated as continuous variables. All the covariates are log-transformed and normal distribution is then assumed during the simulation. Subsequently, the simulated values are converted back into the normal space. The simulated values describing categorical covariates are linked to pre-defined thresholds within the simulated distribution; these thresholds are calculated from the inverse cumulative distribution function of the empirical distribution.

The MVND covariate simulations were performed according to a user-defined function in R (see Electronic supplementary material). This function takes into account the distribution of covariates in the empirical distribution with a tolerance level of 5% for categorical covariates, whereas continuous covariates are retained for values within the range of the real data. These procedures can be adjusted to different acceptance criteria and correspond to simulating from truncated distributions when compared to the empirical (real) covariate distributions. All simulations were based on study design scenarios with group sizes of 100, 1000 and 3500 patients. Since the MVND method consists in the generation of new subjects, these simulated covariates were also compared with the real data. In this evaluation, each simulation was performed in duplicate in order to evaluate the reproducibility of the method.

### Drug-Disease Model for Trough FEV1

In order to evaluate the performance of re-sampling and MVND methodologies in the context of CTS, covariates were simulated in conjunction with a KPD model describing the treatment effects of a bronchodilator on forced expiratory volume 1 sec (FEV1). The analysis was performed using NONMEM 7.1.2 (24, 25).

The changes in FEV_1_ over time were described by an indirect response model, as expressed by the following differential equation:

1 $$ \frac{dFE{V}_1}{dt}= Kin- Kout\times FE{V}_1 $$

where the change in the observed FEV_1_ over time (*d*FEV_1_/*d*t) is controlled by a zero-order process parameterised as a synthesis rate constant (Kin) and first order elimination process (Kout). The disease status at baseline (steady state conditions) was defined by the ratio between Kin and Kout. The disease progression was modelled as a linear decline in the disease status as follows:

2 $$ \mathrm{D}\mathrm{i}\mathrm{s}=\mathrm{I}\mathrm{n}\mathrm{t}\_\mathrm{D}\mathrm{i}\mathrm{s}-\mathrm{Slope}\_\mathrm{D}\mathrm{i}\mathrm{s}\times \mathrm{Time} $$

With

3 $$ \mathrm{D}\mathrm{i}\mathrm{s}=\frac{\mathrm{Kin}}{\mathrm{Kout}} $$

where Dis is the disease status, Int_Dis is the disease status at the start of the clinical trial, and Slope_Dis is the daily decline in Dis due to the disease progression.

As described by Eq. (2), the disease status (Dis) reflects not only the natural disease progression over time, but also time-dependent processes (Eq. 3) associated with the changes in airway function, as determined by spirometry (FEV1). Both processes were found to be affected by an individual patient’s clinical and demographic characteristics.

The KPD model consisted of a nonlinear Emax function in which the maximum effect is proportional to the apparent potency parameter (EDK_50_). Further details on the parameterisation and model validation can be found elsewhere (24). Dropout was not included in this simulation exercise to avoid confounding effects. Each trial was simulated 100 times with a different set of patients.

In spite of the availability of FEV1 response data in the original trial population, we have decided to use a set of simulated responses from the model as reference for the evaluation of the performance of the re-sampling and MVND methods. This choice was made to ensure accurate comparison of results and avoid interference of study-specific aspects (e.g. dropout rate) or model-related issues (e.g. the difference between predicted and observed response) when comparing the different scenarios. The response data from the model are referred in the article as “real data” since they are generated with real covariates. Drug and placebo effect were simulated using the same KPD model as for the reference population. In this case, 100 different data sets were simulated, mimicking the situation observed in 100 different clinical studies with the same inclusion–exclusion criteria, as observed for the empirical distribution. The simulations were performed with a series of *ad hoc* R functions (26) aimed at automating run execution and obtaining basic statistical summaries. The simulations for treatment and placebo arms were performed independently in different data sets to avoid the interference may arise from patient assignment to the different arms.

### MVND to Simulate New Patient Populations

To illustrate the implications of covariate correlations on trial outcome, additional simulation scenarios were considered in which a new patient cohort was created by screening *a posteriori* subsets of patients simulated according to the steps described above. Using the same *ad hoc* functions 3500 patients were selected to generate different stratification levels for disease severity, as compared to the empirical distribution (i.e., from 1:2 to 2:1 for mild and moderate severity).

## Results

**Fig. 2:**
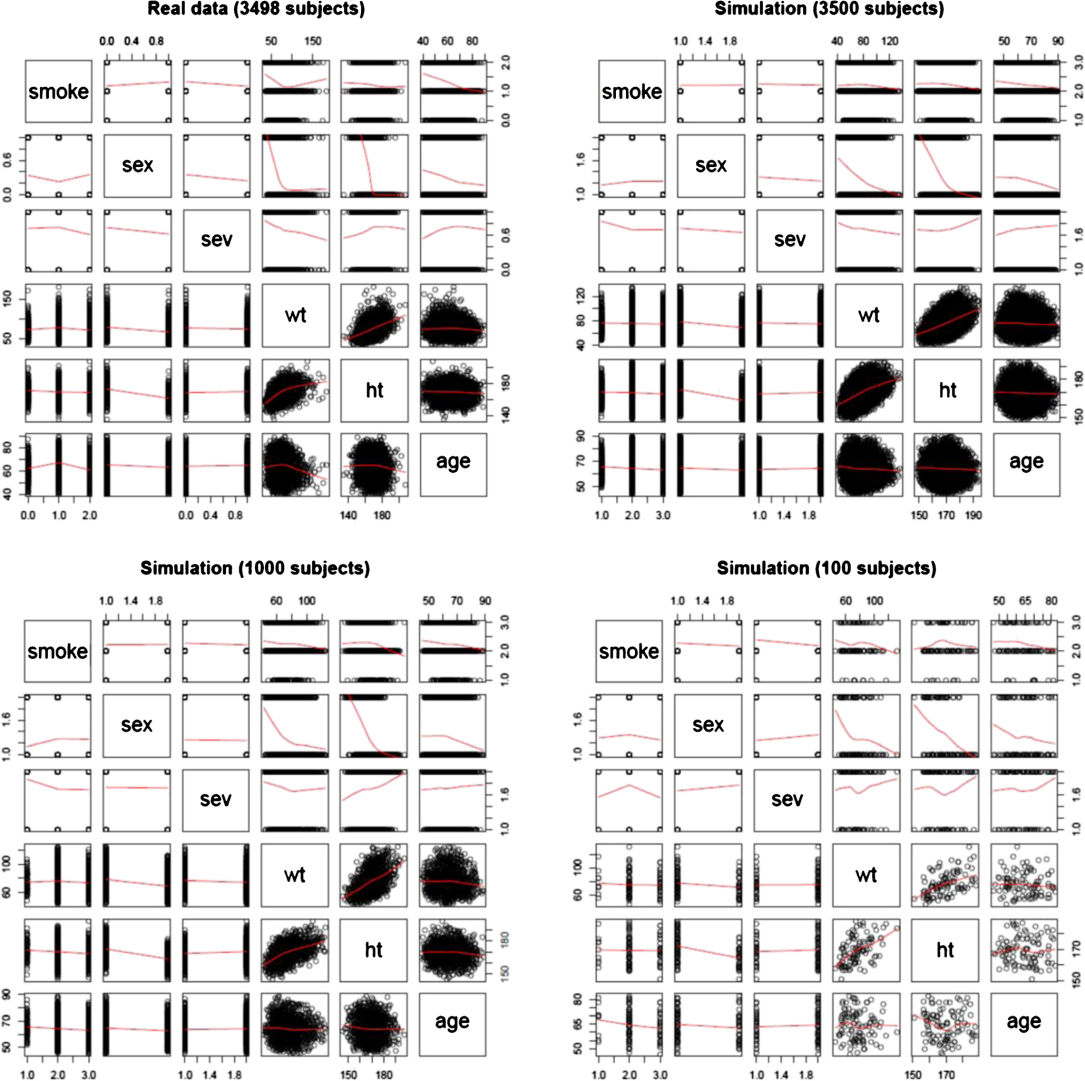
Scatter plot matrix showing the covariate correlation for the real study in which a total of 3498 patients were included (*left upper corner*) and for different simulated patient populations. The simulations included cohorts with 3500 (*right upper corner*), 1000 (*left lower corner*) and 100 (*right lower corner*). The covariates presented in the matrix are respectively: smoking status (smoke), gender (sex), disease severity (sev), weight (wt), height (ht) and age (age).

To assess the performance of the two methods for the simulation of covariates, the first step consisted in verifying the impact of different sample sizes on the covariate distributions and their correlations. Figure 2 depicts the covariate correlations for the real pool of patients and for the different populations simulated with 100, 1000 and 3500 patients.

An initial evaluation of the performance of the MVND method consisted in comparing qualitatively and quantitatively the correlation and proportion profiles of real and virtual patients. In all the simulations scenarios, the correlation between covariates was found to be maintained, for both continuous and categorical covariates. This is warranted also for small sample sizes, although slight deviations were observed for simulations with very low numbers of patients.

**Fig. 3:**
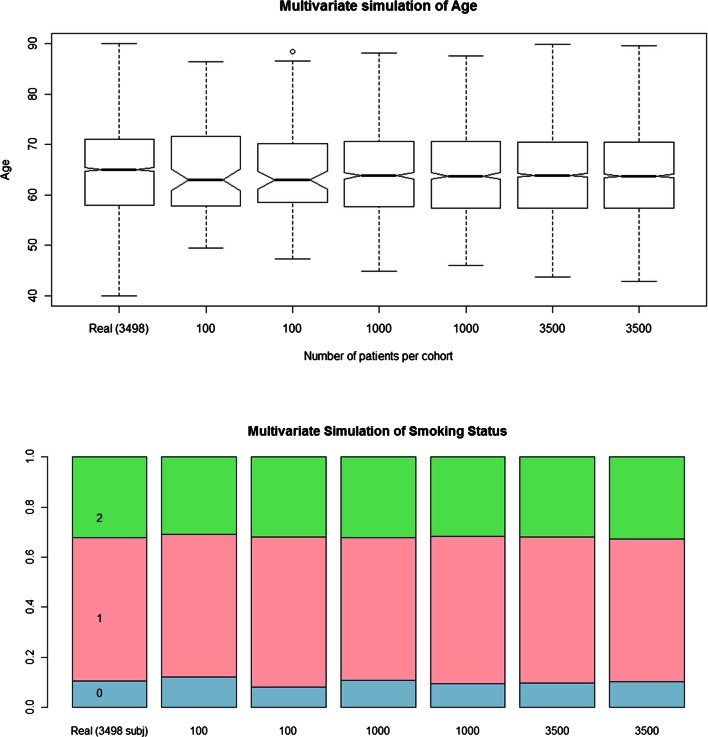
Distributions of age (*top panel*) and proportions of smoking status (*lower panel*) for real and simulated data. Simulated populations comprised different number of patients: 100, 1000 and 3500; each simulation was performed in duplicate.

As shown in Fig. 3 for age and smoking status, a similar pattern is observed for the distribution of the simulated continuous covariates and the proportions for the categorical ones irrespective of sample sizes. Additional details can be found for other continuous and categorical covariates in the Electronic supplementary material (Figure S1 and S2). As depicted in Figs. 3, S1 and S2, the simulated covariates present a median and interquartile range which reflect the covariate distribution in the real population. On the other hand, in some cases the high variability in the original data set has yielded outlier values, which are not captured by the simulations. The concordance between real and simulated data is particularly important to ensure that the simulated data reflect the range of values observed in real life. The proportion of the categorical covariates also reflects the structure of the original pool of patients, showing that this technique can be used to reproduce the patient stratification used in the real trial.

### CTS with Re-Sampling and MVND Patient Generation

**Fig. 4:**
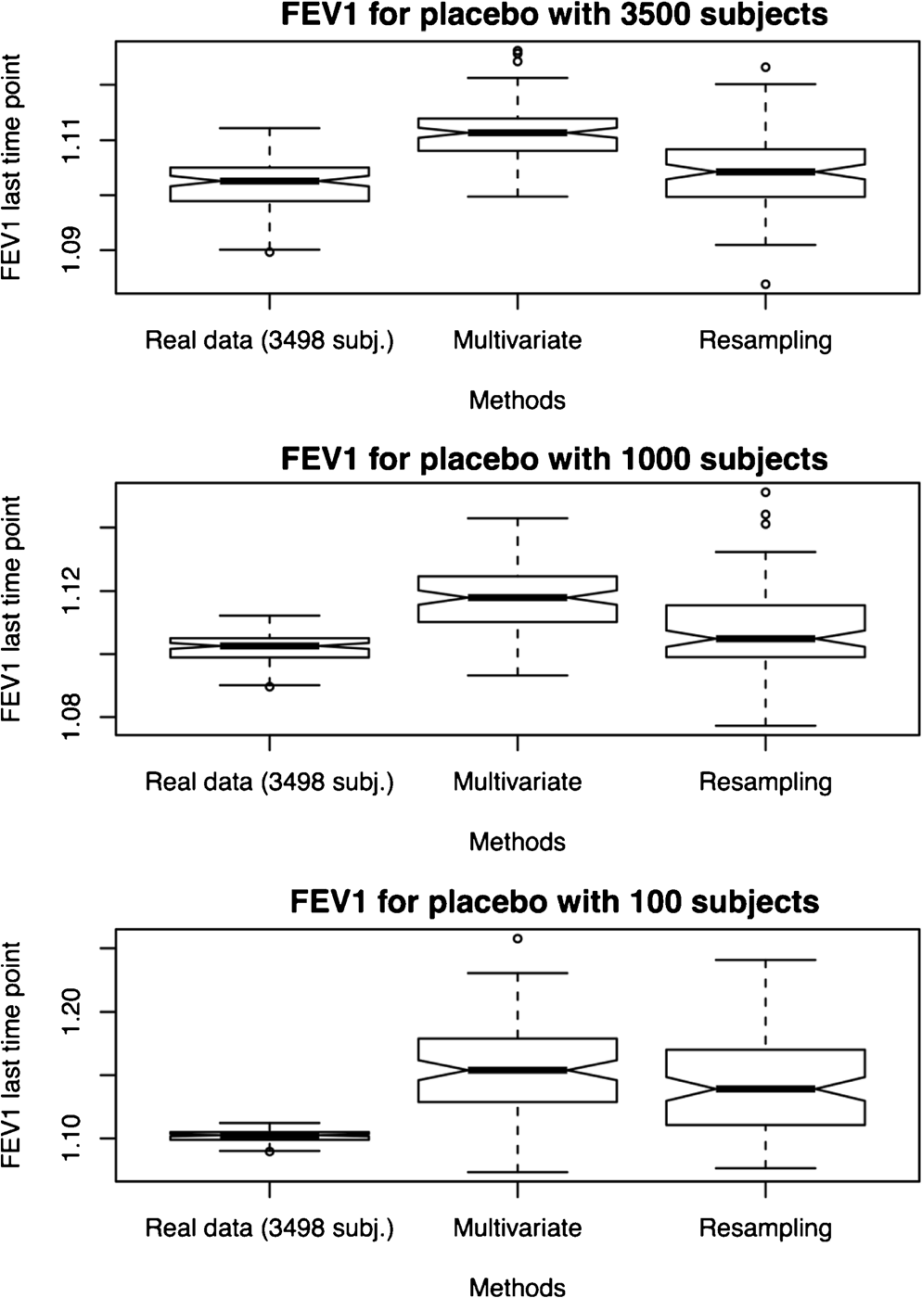
Trough FEV1 value reached at the end of the trial for the placebo arm. Scenarios with different number of patients were evaluated: 100 (*lower panel*), 1000 (*middle panel*) and 3500 (*upper panel*). The differences in the predicted response obtained by multivariate distribution reveals the role of underlying covariate correlations, which are not considered when re-sampling techniques by bootstrapping are used. Sample size has a clear effect on the distribution of the predicted results, irrespective of the simulation method.

Figure 4 shows the results for FEV1 at the end of the trial based on the re-sampling (bootstrapping) and MVND methods. The former reflects the impact of covariate correlations in the original population whereas the latter relies on the estimated multivariate distribution. No formal statistical hypothesis test is required to conclude that different results are predicted for trough FEV1 values, depending on the method used and characteristics of the simulated population. The magnitude of such differences may vary with the sample size. As expected the simulated FEV1 vs. time profiles were similar to the original trial when considering comparable or exchangeable patient populations (i.e., bootstrapping). The similarities in the results hold also after varying sample size. By contrast, multivariate methods also generate realistic covariate distributions, but provide insight into the impact of the interaction between covariates (i.e., covariate correlations) in the overall population. These differences unravel variation which is often assigned to mere randomness when comparing clinical trial results in the presence of covariate effects.

**Fig. 5:**
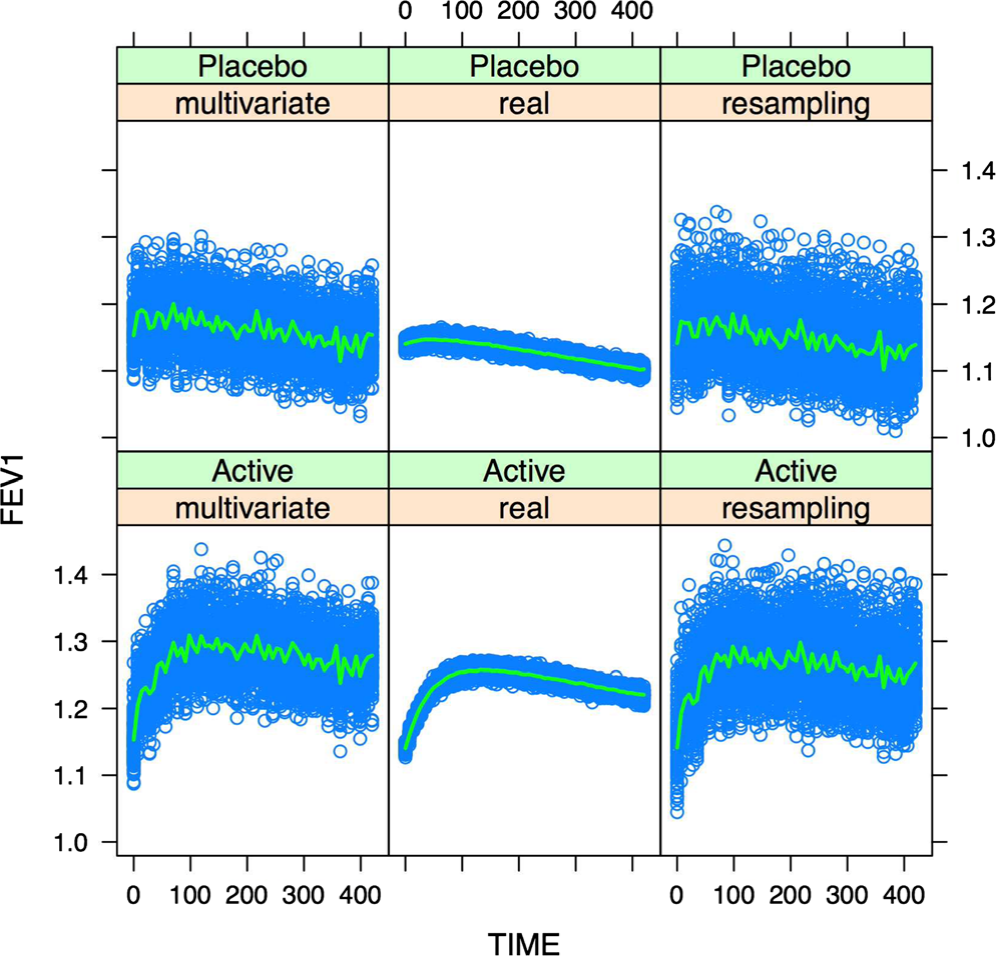
Simulation of 100 clinical trials using the multivariate distribution or re-sampling method (each trial has 100 COPD patients per arm). The results are compared with the findings obtained with the same model for the real population of 3498 patients. The *blue dots* represent the medians of the 100 trials, while the *green line* represents the median of the medians.

**Fig. 6:**
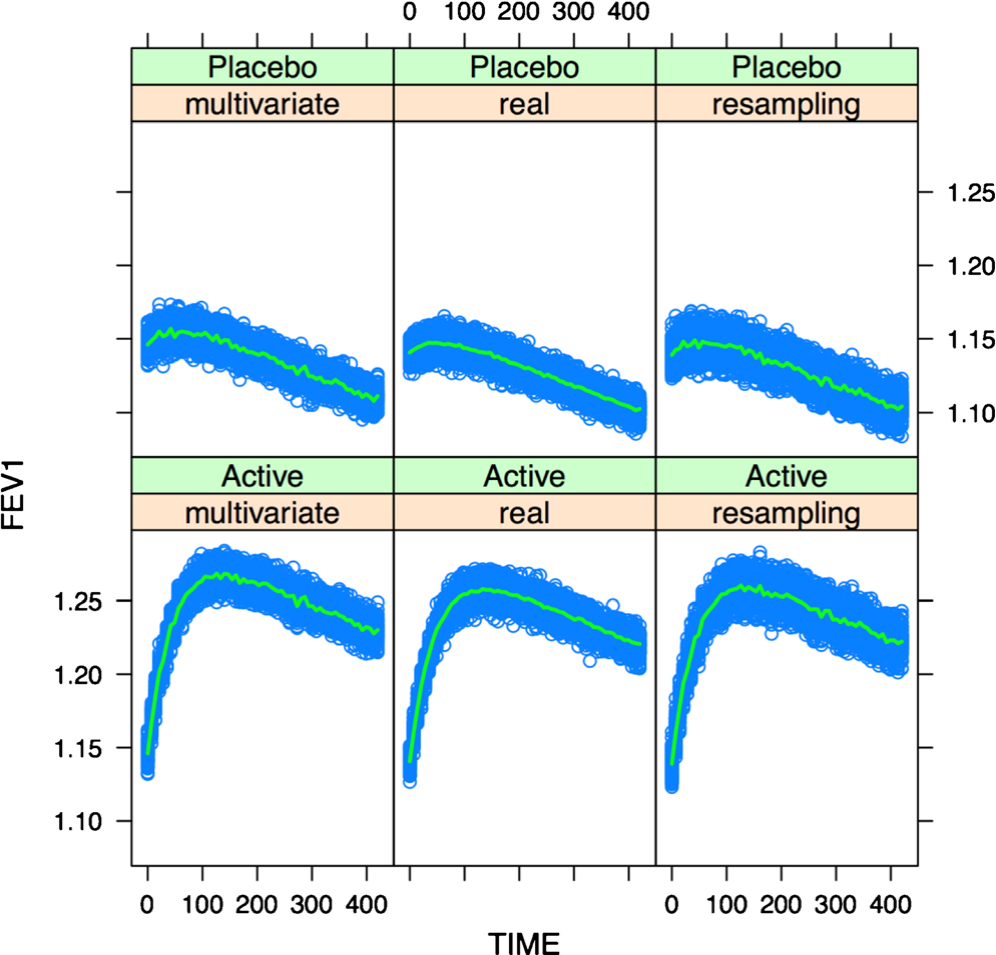
Simulation of 100 clinical trials using the multivariate distribution or re-sampling method (each trial has 3500 COPD patients per arm). The results are compared with the findings obtained with the same model for the real population of 3498 patients. The *blue dots* represent the medians of the 100 trials, while the *green line* represents the median of the medians.

Given the presence of time-dependent covariate effects, a comprehensive summary of the two methods is provided in Figs. 5, 6 and 3S, in which FEV1 changes in 100 clinical trials are depicted over the period of 1 year. It should be underlined that despite good prediction of the mean profiles for FEV1, the variability of the simulated data was significantly higher than the real for scenarios based on smaller sample sizes (Figs. 5 and 3S). By contrast, this issue was not evident when results from scenarios with similar sample sizes are compared with each other (Fig. 6).

Overall, it appears that the trials simulated with the multivariate distribution present slightly smaller variability as compared with those obtained by discrete re-sampling.

### MVND to Simulate New Patient Populations

The main advantage of MVND method compared to discrete re-sampling is the possibility to create a new pool of patients across a different range of values or stratification levels, whilst ensuring the correlation structure and/or colinearity are maintained.. An example of this feature is presented in Figure 4S (Electronic supplementary material), which shows a new study population stratified by disease severity using a different proportion of patients, as compared to the original patient pool. The FEV1 vs. time profile is similar to the original study, but the FEV1 at baseline is higher for the simulated population.

## Discussion

CTS is a powerful resource for the prediction of the outcome of hypothetical clinical trials. However to be effective as a design tool, it is essential to ensure that the patient populations used in the simulation process reflect real patients and that covariate correlations are well described. Particularly important is the covariate distribution defining the correlation between patient demographic and disease characteristics, given that they are often related to the study outcome (12, 27). Such correlations have so far been overlooked in previous investigations in which covariate effects are treated as structural parameters in pharmacokinetic or pharmacokinetic-pharmacodynamic models. The need for identifying covariate correlations and incorporating them into a matrix for the simulation of virtual patients is disregarded in most publications (18). Our investigation has shown the practical implications of discrete re-sampling and MVND for the creation of a virtual patient population.

The concept of re-sampling data – more commonly referred to as bootstrapping – has been in use for more than four decades. Bootstrapping has been shown to have considerable theoretical advantages when it is applied to non-Gaussian data (28). However, its application in CTS presents an important limitation, which is crucial for prospective evaluation of drug response in drug development, which often involves changes in protocol design, doses and most importantly different inclusion and exclusion criteria, making the patient population in a trial not necessarily exchangeable with the subsequent one, as for instance during Phase II and Phase III studies (29, 30). By contrast, the MVND technique allows simulation of new vectors of covariates, which can differ from the ones present in the initial pool of subjects. A limitation of this methodology has been however its use for continuous variables only. We have implemented the procedure described by Tannenbaum *et al.* (20) into an R function which allows the application of such method for the simulation of continuous as well as categorical covariates (31).

### Advantages and Limitations

Historically in pharmacometric research, bootstrapping and other re-sampling techniques have been applied as a statistical tool for model diagnostics and more specifically parameter estimation, i.e., to assess its accuracy and bias. Some of the elementary uses of bootstrapping include the calculation of confidence intervals, hypothesis testing, linear regression, and correlations when exploring the association between variables (32). The technique may also be useful for analysing smallish expensive-to-collect data sets, where prior information is sparse, distributional assumptions are unclear, and where further data may be difficult to acquire (33). These applications do not address the requirements for characterising the impact of covariate effects on a prospective, potentially different population. The increasing focus on personalised medicines and the use of adaptive protocol designs in clinical research impose the need for trial models in which patient population characteristics are clearly reflected in a simulation scenario, especially when making predictions from one group to another (34). From a statistical point of view, such differences in experimental conditions and varying population characteristics can limit the predictive performance of the models used in clinical trial simulations.

In fact, the possibility to evaluate the impact of greater heterogeneity and variability in the study population, as compared to randomised controlled trials, is critical for the use of clinical trial simulations as a quantitative tool for regulatory and clinical decision making. The ability to make inferences and draw conclusions about treatment response in a larger population involves assumptions about the representativeness of the cohort for the ‘target’ population (35, 36). In conjunction with the appropriate model parameterisation, it may also provide the basis for identifying the physiological and clinical mechanisms underlying statistical correlations (37). It should be noted, however, that when using MVND all the covariates are assumed to follow the same distribution, in this particular case, the log-normal distribution. A potential limitation of this assumption is that the multivariate distribution is based on only one vector of covariates per subject, which restricts the simulations to time invariant covariates or baseline values.

### COPD as a Paradigm Population

The assessment of response to an intervention should be based on sensitive clinical measures or endpoints that reflect differences in disease severity and/or patient population characteristics. This has been previously illustrated for HIV infection, where CD4 counts and mRNA viral load are correlated with clinical status, antiviral treatment effect, and risk of AIDS (38). Despite ongoing efforts on the evaluation of novel treatments for COPD, little has been made to characterise how differences in patient characteristics, i.e., inclusion and exclusion criteria, correlate with the clinical status and overall treatment response (21, 22).

Our investigation shows that covariate correlations, as defined by the underlying correlation structure, can have clear implications for the outcome of a trial (24, 39). In addition, we have found that for small sample sizes, simulated FEV1 profiles show larger variability as compared to the real data. This is a natural consequence of the smaller sample size of the simulated scenarios (with MVND and re-sampling). On the other hand, when the sample size is comparable to the sample size of the empirical distribution, the FEV1 profiles mimic those of the real population. Another point worth mentioning is that trials simulated with the MVND method show lower variability as compared to those using re-sampling. This may be due to the inclusion of distribution boundaries in the simulation step with the MVND (40). In fact, to avoid unrealistic covariates values, continuous covariate values were retained if within the range of real data. Likewise, for categorical covariates a 5% tolerance was applied as acceptance criterion for differences in the covariate distribution in the simulated and real data sets. In addition, one should bear in mind that the values simulated by re-sampling are driven by the available data, which leads to empirical distributions that are closer or similar to the source data. By contrast, the data simulated with the MVND include a well-defined variance-covariance structure under the assumption that all covariates are log-normally distributed and this distribution propagates across all subgroups (e.g. male and female).

### Perspectives

In summary, CTS needs to account for the main sources of variability affecting the variables of interest during an actual trial. In contrast to traditional re-sampling techniques, the MVND method allows simulation of new patient pools in which patient distributions are generated from a pre-defined covariate correlation model, irrespective of inclusion and exclusion criteria. Moreover, since new covariate vectors can be generated, it is possible to evaluate patient distributions which may not have been tested experimentally. In spite of current views on the role of genotypical and phenotypical markers as the basis for stratification in clinical trials (41, 42), our findings suggest that stratification procedures are also necessary in clinical trial simulations whenever moderate and strong covariate effects have been shown to affect pharmacokinetics and pharmacodynamics.

## Electronic supplementary material

Supplemental material (PDF 372 kb)

## ABBREVIATIONS

COPD: Chronic obstructive pulmonary disease
CTS: Clinical trial simulation
Dis: Disease status
EDK_50_: Apparent potency
Emax: Maximum effect
FEV_1_: Forced expiratory volume in 1 second
Int_Dis: Disease status at the start of the clinical trial
Kin: Zero-order synthesis rate constant
Kout: First order elimination process
KPD: Kinetic-pharmacodynamic
MVND: Multivariate normal distribution
PKPD: Pharmacokinetic-pharmacodynamic
Slope_Dis: Daily decline in disease status
