# Supplementary material for: Generating Virtual Patients by Multivariate and Discrete Re-Sampling Techniques
Source: Pharm Res. 2015 May 21;32(10):3228–37. doi: 10.1007/s11095-015-1699-x (PMC4577546; doi:10.1007/s11095-015-1699-x)
Supplement: Supplementary file 1 — (PDF 372 kb) [file 11095_2015_1699_MOESM1_ESM.pdf]

Supplemental material

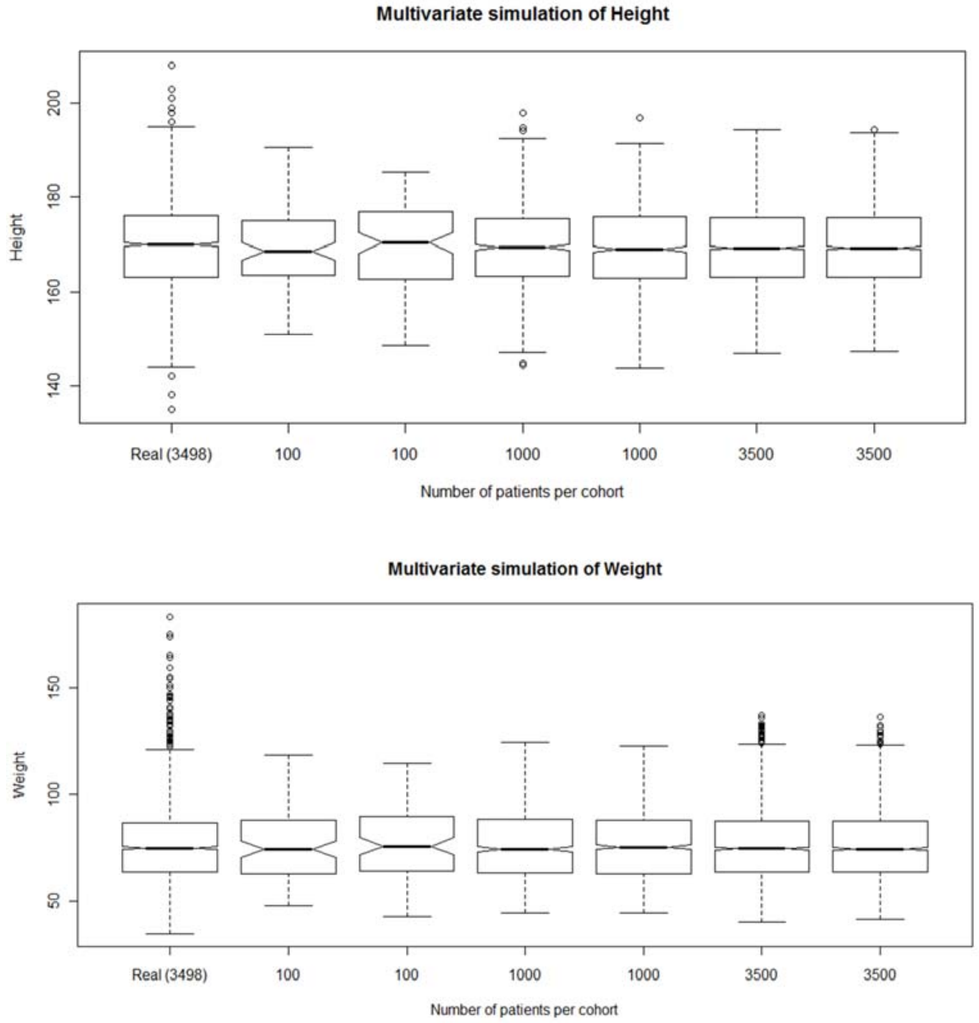

Figure 1S: Distributions of height (upper panel) and weight (lower panel) for real and simulated data. Simulated populations comprised different number of patients: 100, 1000 and 3500; each simulation scenario was performed in duplicate.

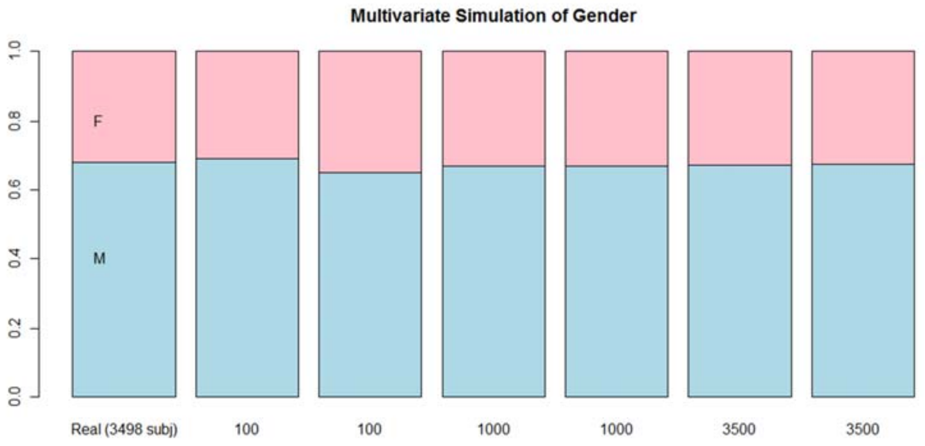

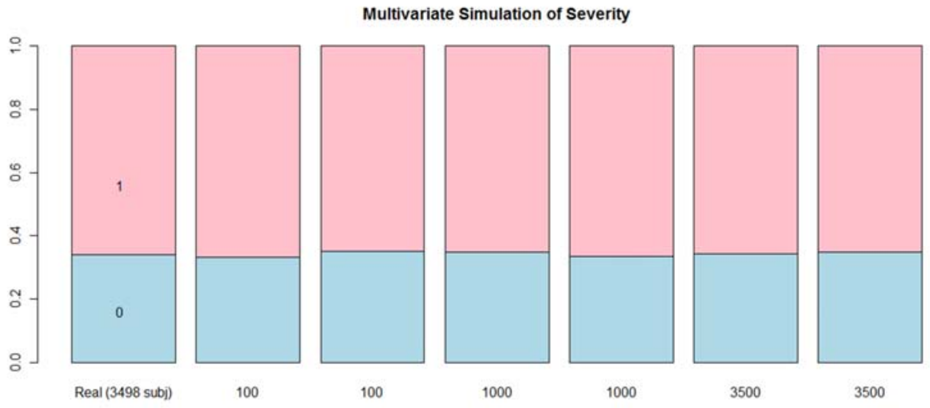

Figure 2S: Proportions of gender (upper panel) and severity (lower panel) for real and simulated data. Simulated populations comprised different number of patients: 100, 1000 and 3500; each simulation scenario was performed in duplicate.

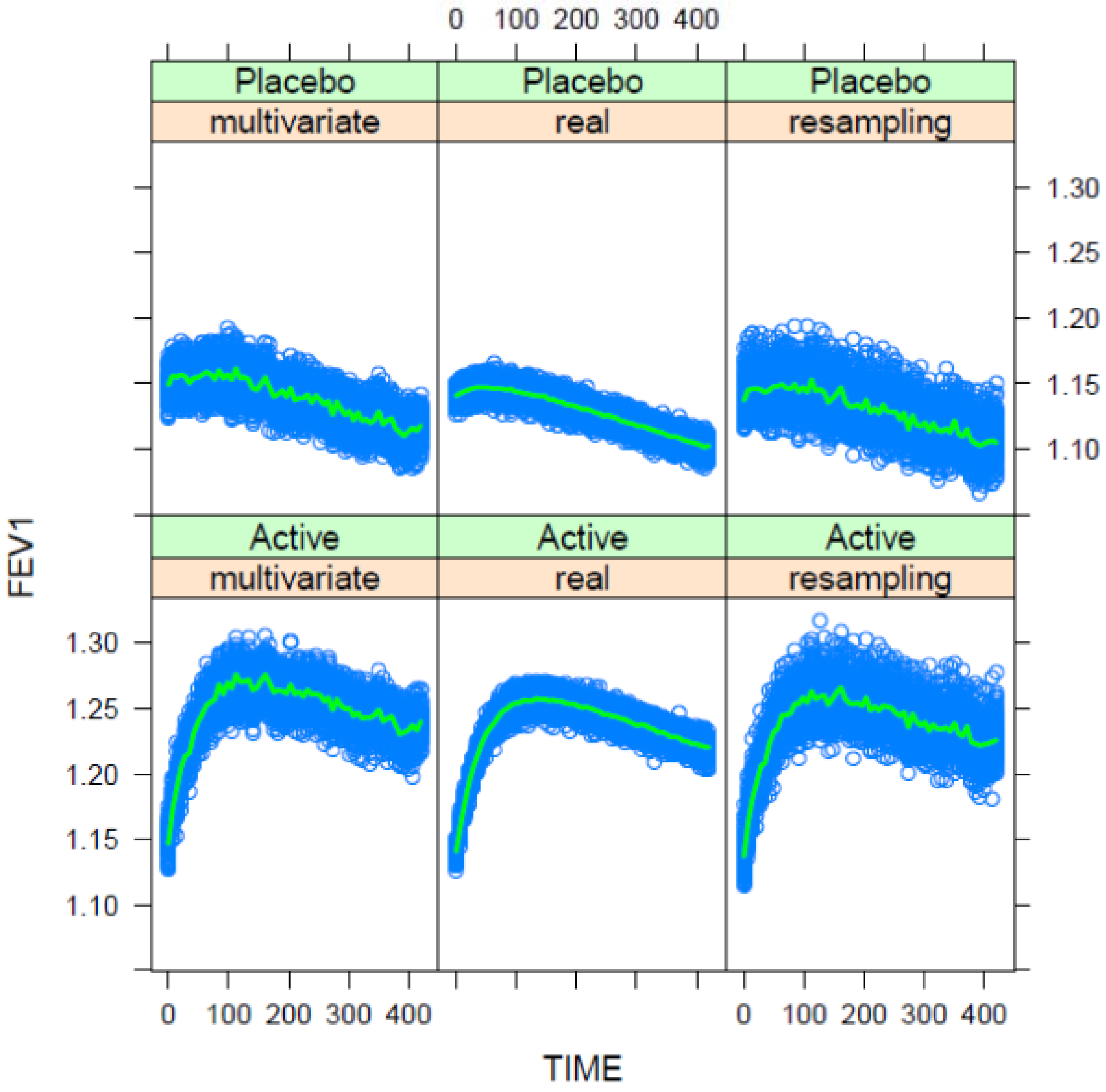

Figure 3S: Simulation of 100 clinical trials using the multivariate distribution or re-sampling method (each trial has 1000 COPD patients per arm). The results are compared with the findings obtained with the same model for the real population of 3498 patients. The blue dots represent the medians of the 100 trials, while the green line represents the median of the medians.

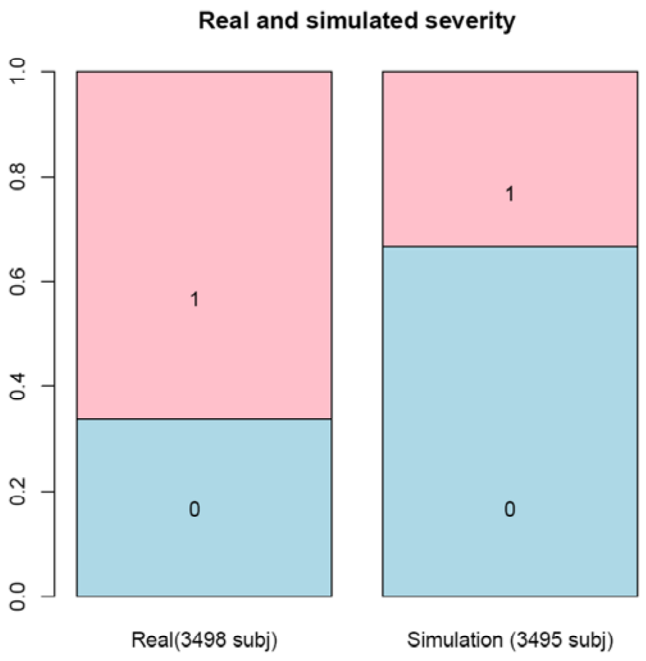

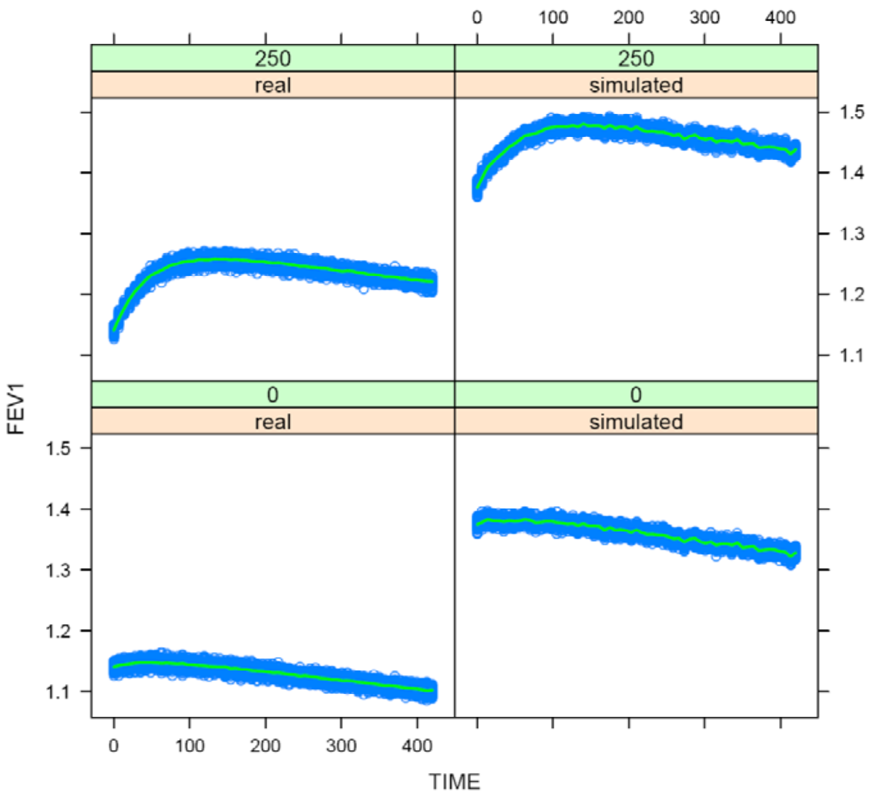

Figure 4S: Simulation of 100 clinical trials with different stratification rules, as defined by the proportion of severe patients in a trial (from 1:2 to 2:1 for mild and moderate severity).
